# Guidance for the Clinical Use of the Breast Cancer Polygenic Risk Scores

**DOI:** 10.3390/cancers17071056

**Published:** 2025-03-21

**Authors:** Peeter Padrik, Neeme Tõnisson, Tone Hovda, Kristine Kleivi Sahlberg, Eivind Hovig, Luís Costa, Gonçalo Nogueira da Costa, Inna Feldman, Filipa Sampaio, Sander Pajusalu, Kristiina Ojamaa, Kersti Kallak, Ave-Triin Tihamäe, Laura Roht, Tiina Kahre, Anni Lepland, Siim Sõber, Krista Kruuv-Käo, Madli Tamm, Jajini Varghese, Dafydd Gareth Evans

**Affiliations:** 1 Clinic of Hematology and Oncology, Tartu University Hospital, 50603 Tartu, Estonia; kristiina.ojamaa@kliinikum.ee (K.O.); kersti.kallak@kliinikum.ee (K.K.); 2 OÜ Antegenes, 50603 Tartu, Estonia; siim.sober@antegenes.com (S.S.); krista.kruuv-kao@antegenes.com (K.K.-K.); 3 Institute of Genomics, University of Tartu, 51010 Tartu, Estonia; neeme.tonisson@ut.ee (N.T.); madli.tamm@ut.ee (M.T.); 4 Genetics and Personalized Medicine Clinic, Tartu University Hospital, 50406 Tartu, Estonia; sander.pajusalu@kliinikum.ee (S.P.); laura.roht@kliinikum.ee (L.R.); tiina.kahre@kliinikum.ee (T.K.); anni.lepland@kliinikum.ee (A.L.); 5 Department of Radiology, Vestre Viken Hospital Trust, 3004 Drammen, Norway; tone.hovda@vestreviken.no; 6 Department of Research and Innovation, Vestre Viken Hospital Trust, 3004 Drammen, Norway; kristine.sahlberg@vestreviken.no; 7 Institute for Clinical Medicine, Faculty of Medicine, University of Oslo, 0424 Oslo, Norway; 8 Department of Tumor Biology, Institute of Cancer Research, Oslo University Hospital, 0424 Oslo, Norway; ehovig@ifi.uio.no; 9 Hospital de Santa Maria, Centro Hospitalar Universitário Lisboa Norte, 1649-028 Lisbon, Portugal; luis.costa@chln.min-saude.pt (L.C.); goncalo.costa@ulssm.min-saude.pt (G.N.d.C.); 10 Instituto de Medicina Molecular-João Lobo Antunes, Faculdade de Medicina, Universidade de Lisboa, 1649-028 Lisbon, Portugal; 11 Faculdade de Medicina, Universidade de Lisboa, 1649-028 Lisbon, Portugal; 12 Department of Public Health and Caring Sciences, Uppsala University, 753 10 Uppsala, Sweden; inna.feldman@regionuppsala.se (I.F.); filipa.sampaio@uu.se (F.S.); 13 Institute of Clinical Medicine, University of Tartu, 51010 Tartu, Estonia; 14 Clinic of Surgery, Tartu University Hospital, 50406 Tartu, Estonia; ave-triin.tihamae@kliinikum.ee; 15 Royal Free London NHS Trust, UCL Division of Surgery and Interventional Sciences, London NW3 2QG, UK; jajini.varghese1@nhs.net; 16 Manchester Centre for Genomic Medicine, Division of Evolution, Infection, and Genomic Sciences, University of Manchester, Manchester M13 9PL, UK; gareth.evans@mft.nhs.uk

**Keywords:** breast cancer, polygenic risk score, genetic predisposition, prevention, screening, personalised medicine

## Abstract

**Background/Objectives**: Polygenic risk scores (PRSs) have been extensively studied and are increasingly applied in healthcare. One of the most studied and developed areas is predictive medicine for breast cancer, but there is no wider consensus on the indications for the clinical use of PRSs for breast cancer. This current guidance endeavours to articulate the scientific evidence underpinning the clinical utility of PRSs in stratifying breast cancer risk, with a particular emphasis on clinical application. **Methods**: This guidance has been prepared by a group of experts who have been active in breast cancer PRS research and development, combining a review of the evidence base with expert opinion for indications for clinical use. **Results**: Based on data from various studies and existing breast cancer prevention and screening services, the indications for clinical use of breast cancer PRSs can be divided into the following scenarios: (1) Management of cancer-free women with a family history of cancer; (2) individual personalised breast cancer prevention and screening in healthcare services; and (3) breast cancer screening programs for more personalised screening. **Conclusions**: The integration of PRSs into clinical practice enables healthcare providers to deliver more accurate risk assessments, personalised prevention strategies, and optimised screening programmes, thereby improving patient outcomes and enhancing the effectiveness of breast cancer care. PRS testing represents a novel component in clinical breast cancer risk assessment, supporting a personalised, risk-based approach to breast cancer prevention and screening.

## Simple Summary

Breast cancer is a significant health concern, and finding effective ways to predict and prevent it is crucial. This review focuses on polygenic risk scores (PRSs), which are tools that use genetic information to assess a person’s risk of developing breast cancer. While PRSs have been studied extensively, there is no clear agreement on how and when it should be used in healthcare. Our work aims to provide guidance on how PRSs can help identify women at higher risk of breast cancer and tailor their prevention and screening accordingly. By integrating PRSs into healthcare, doctors can offer more personalised care, such as targeted screening and prevention strategies, based on an individual’s genetic risk. This approach has the potential to improve patient outcomes, making breast cancer prevention and care more effective and focused.

## 1. Introduction

Polygenic risk scores (PRSs) have been extensively studied and are increasingly applied in healthcare. One of the most researched and developed areas for this is predictive medicine for breast cancer, but so far there is no wider consensus on the indications for the clinical use of PRSs for breast cancer. The American College of Medical Genetics and Genomics (ACMG) has published the statement about the clinical application of PRSs with points to consider in applications [1]. The authors state that although being rapidly incorporated into health care, there are currently no clinical guidelines available for the use of this technology. This current guidance endeavours to articulate the scientific evidence underpinning the clinical utility of PRSs in stratifying breast cancer risk, with a particular emphasis on clinical application. It delineates pertinent scenarios wherein its clinical application is relevant and deliberates on the methodologies through which these principles can be pragmatically instituted within a clinical environment. This guidance has been prepared by a group of experts who have been active in breast cancer research, including PRS research and other relevant projects, as well as in clinical use. The AnteNOR and BRIGHT projects have investigated and clinically developed the application of PRSs in personalised breast cancer prevention and screening [2,3].

To the extent that the contexts of cancer prevention and PRSs can be different in different healthcare systems, we will discuss it primarily in the context of European healthcare systems.

## 2. Background

### 2.1. Breast Cancer Prevention and Screening

Breast cancer is the most commonly diagnosed cancer among women worldwide and the leading cause of cancer-related deaths in women, with approximately 2.3 million new cases and 660,000 deaths annually [4]. Breast cancer morbidity and mortality can be reduced through primary and secondary prevention.

Primary breast cancer prevention involves lifestyle modifications such as a healthy diet, weight control, regular exercise, and limiting alcohol intake and prolonged breastfeeding, alongside medical interventions including hormonal preventive medication and risk-reducing surgery in very high-risk cases, all tailored to individual risk factors and developed in consultation with healthcare providers [5].

Secondary breast cancer prevention with mammography screening reduces mortality risk from breast cancer by 20–30% [6,7,8]. Breast cancer incidence rates vary significantly across different age groups and populations globally; therefore, we describe the background in the European context. European breast cancer screening programs typically target women aged 50 to 69 years and are mostly based on age only [9,10]. The primary method used is mammography, performed biennially or triennially in the United Kingdom. Some countries also include women aged 40 or 45 to 74 in their screening programs, recognizing the potential benefits in slightly younger and older populations. European guidelines on breast cancer screening and diagnosis from the “European Commission Initiative on Breast and Colorectal Cancer” recommends regular screening for asymptomatic women with an average risk of breast cancer screening from age 45 [10]. However, this approach fails to consider the substantial variation in individual breast cancer risk among women. It overlooks younger women who may have an elevated risk, as well as those over 45 or 50 who could benefit from enhanced screening due to higher risk levels. The traditional one-size-fits-all approach to breast cancer screening does not account for individual variations in risk factors such as genetic predisposition, family history, breast density, and lifestyle factors. Risk-stratified screening aims to tailor screening strategies to individual risk profiles, enhancing the benefits while minimizing the harms. An alternative to age-based screening is risk-based screening, where individual risk assessments guide screening recommendations [11,12].

It has been shown that hereditary factors account for approximately one-third of overall breast cancer risk [13]. Therefore, genetic predisposition followed by genetic risk assessments are an extremely important component in risk-based, or personalised, breast cancer prevention. Genetic factors encompass rare monogenic pathogenic variants (MPVs) in high- and moderate-risk cancer predisposition genes. Their impact is significant enough to justify testing for monogenic variants. However, these rare MPVs are connected with only a small proportion (5–10%) of breast cancer cases [14]. Indeed, only around 1.7% of people and 5.7% of those with breast cancer currently are known to carry MPVs in the 12–13 actionable breast cancer genes conferring at least a reported two-fold risk [15]. A significant proportion of breast cancer risk variation is attributed to common single-nucleotide polymorphisms (SNPs) located outside high- and moderate-risk genes. These SNPs have been identified through genome-wide association studies (GWASs) [16,17]. A polygenic risk score (PRS) represents the cumulative impact of multiple breast cancer susceptibility SNPs. While individual SNPs may confer only a modest risk, their combined effect can be considerable. Additionally, genetic susceptibility may also be influenced by family history, even in the absence of a known MPV or PRS data.

### 2.2. Monogenic Breast Cancer Risk

Certain monogenic pathogenic variants (MPVs) in individual genes result in a significantly higher predisposition to breast cancer. Recent analyses have specified MPVs in the genes *ATM*, *BARD1*, *BRCA1*, *BRCA2*, *CDH1*, *CHEK2*, *PALB2*, *RAD51C*, *RAD51D*, *TP53*, *PTEN*, and *STK11*, all associated with higher breast cancer risk levels [18,19,20,21].

Testing for MPVs requires panel sequencing of selected genes or sequencing of the entire exome or genome. Indications for MPV testing in healthy individuals, but also in breast cancer patients, are usually defined by international and national guidelines using family cancer history and/or age at onset and cancer pathology [22,23,24,25,26]. For instance, in the UK all patients with triple-negative breast cancer qualify for testing [27].

Carriers of MPVs associated with an increased risk of breast cancer are recommended to undergo more intensive surveillance and may also be offered additional preventative options. The specifics of how carriers are followed can vary based on individual risk factors, family history, and the type of variant.

### 2.3. Polygenic Breast Cancer Risk

A substantial portion of breast cancer risk variation is linked to common SNPs located outside high- and moderate-risk genes [16]. In general, a PRS estimates an individual’s likelihood of developing a specific condition, based on the weighted contribution of risk-associated single-nucleotide variants identified through GWASs [28]. A breast cancer PRS reflects the cumulative impact of these susceptibility variants, which have been shown to effectively stratify individual breast cancer risk [17,29,30,31,32,33].

PRSs are the strongest independent risk factor for breast cancer development on a population basis [34,35], outweighing MPVs. Breast cancer PRSs highlight variations in genetic risk and serve as a foundation for developing personalised screening programmes that consider individual genetic susceptibility [36]. In particular, in contrast to MPV testing, they identify a low-risk population. Modelling studies indicate that incorporating risk profiles into preventive strategies may lead to both cost savings and improved health outcomes [37,38,39]. A high-risk assessment may also justify the use of hormonal preventive medication [40].

Assessment of the performance of PRSs is commonly performed using hazard ratios (HRs) or odds ratios (ORs) for an increment of one standard deviation in the score and the area under the receiver operating characteristic curve (AUC) [41]. Breast cancer PRSs alone can be considered to exhibit modest discrimination with AUC around 0.6–0.65 [42]; however, when considering PRSs’ use in the clinical context of asymptomatic women, the added information provided by PRSs has the potential to detect a large portion of the population at increased risk of breast cancer [43].

When modelling breast cancer risk, it is common to report HRs for the top quintile, decile, or the highest 5%, 2%, or 1% of PRS distributions, compared to an average or low PRS category. Additionally, breast cancer incidence rates across PRS categories provide valuable insights. In a landmark study, Mavaddat with colleagues developed several PRSs for breast cancer using the largest available genome-wide association dataset and validated them empirically in prospective studies involving individuals of European ancestries [29]. The most effective PRS, comprising 313 SNPs, demonstrated an ORs of 1.61 (95% CI: 1.57–1.65) across 10 prospective studies, with an AUC of 0.630 (95% CI: 0.618–0.651). Women in the highest 1% of the PRS distribution had a lifetime breast cancer risk of 32.6%, with a 4.37-fold increased risk of developing oestrogen receptor-positive disease and a 2.78-fold increased risk of oestrogen receptor-negative disease compared to those in the middle quintile. Conversely, those in the lowest 1% of risk had 0.16- and 0.27-fold risks, respectively. Goodness-of-fit tests confirmed that this PRS was well calibrated and accurately predicted disease risk, particularly at the extremes of the distribution. The study concluded that this breast cancer PRS is a robust and reliable risk predictor with potential to enhance prevention programmes [29].

The development of a clinical-grade level breast cancer PRS test and the clinical implementation outside of research settings have been described by Padrik et al. [44]. The process aimed to develop a clinical-grade PRS test suitable for risk-stratified breast cancer screening, accompanied by clinical implementation guidelines. In the initial phase, previously published PRS models for breast cancer risk prediction were reviewed and validated using data from the Estonian Biobank and UK Biobank. The most effective model was selected based on prevalence data and independently validated in both incident datasets. This optimal PRS, incorporating 2803 SNPs (PRS2803), achieved a concordance index (C-index) of 0.656 (SE = 0.05) in a Cox regression model assessing breast cancer status. The associated HR was 1.66 in the Estonian Biobank and 1.56 in the UK Biobank data. The PRS effectively stratifies individuals, identifying those with more than a three-fold increased risk. Following absolute risk simulations, risk-based recommendations were developed, calibrations to African, East-Asian, South-Asian, and mixed populations were performed based on UK Biobank data and the test was registered as a CE-marked in vitro device (the AnteBC test) and implemented into clinical practice [44]. The test performance data were also additionally analysed using Norwegian population genetic data, confirming the risk-separation performance [45].

PRSs differ from monogenic tests, which target specific genes to detect variants with substantial individual effects. In contrast, PRSs assess a cumulative risk of multiple genetic loci. Unlike monogenic disorders, complex multifactorial diseases necessitate non-family-based risk assessment approaches, making PRSs a valuable and necessary tool. In clinical practice, it is important to consider the impact of both types of genetic predisposition.

### 2.4. Possibilities to Combine Breast Cancer PRSs with Other Risk Factors

Several integrated risk prediction models combine traditional risk factors, including demographic characteristics, reproductive history, menopausal status, family history, prior biopsies, mammographic density, and carrier status of MPVs and PRSs [35,46,47,48,49]. For comprehensive breast cancer risk predictions, PRS test information can be used within combined risk models such as CanRisk or Tyrer–Cuzick [48,50]. In these models, the use of other known risk factors in combination with PRSs has been shown to enhance the prediction of combined models [42,51,52].

Lee et al. demonstrated that a combined risk model CanRisk with a PRS, family history, breast density, and other risk factors could identify approximately 13% of the population as being at moderate or high risk of developing breast cancer, based on the UK National Institute for Health and Care Excellence (NICE) guidelines [22,35]. Using the Tyrer–Cuzick model, this can rise to as high as 20% [51]. As expected, the variation in risk is greatest when including all risk factors in the model [35].

A PRS alone has been shown to predict breast cancer risk more accurately than existing clinical models in individuals of European descent [53]. Van den Broek et al. evaluated the clinical utility of a PRS alongside a first-degree family history of breast cancer to guide screening decisions for women aged 30–50 years [54]. Their findings suggested that incorporating PRSs and family history could help refine screening strategies before the age of 50, potentially reducing breast cancer mortality among women at high risk due to genetic susceptibility. Additionally, an analysis by Wolfson et al. concluded that population-wide breast cancer screening programmes aiming to stratify women by genetic risk should prioritise PRSs over rarer but highly penetrant variants or family history [34]. The PROCAS study also showed that the main factor influencing risk stratification was increasing the number of SNPs in the PRS rather than adding a gene panel [55]. The PRS was most predictive factor for identifying women at high risk, while family history was the weakest.

The results of the clinically available AnteBC test (PRS2803, OÜ Antegenes, Estonia) can be used in the CanRisk combined breast cancer risk assessment model by entering the z-score from the AnteBC test report and the alpha value of 0.437 or in Tyrer–Cuzick model using the non-logarithmic OR.

## 3. Utilising Breast Cancer Polygenic Risk Scores in Clinical Practice

Based on published research evidence, breast cancer PRSs have become an increasingly relevant tool in the landscape of breast cancer risk-stratified prevention and screening. Here are the primary clinical scenarios where the use of PRSs can be particularly impactful:

(1) Management of healthy women with a family history of cancer in hereditary cancer clinics [56,57,58,59,60,61,62,63,64,65,66,67];
(2) Individual personalised breast cancer prevention and screening in healthcare services [44,51];
(3) Breast cancer screening programs to make screening more precise and effective [34,54,68,69,70,71].

If non-genetic risk data are available, and the process is feasible, then it is possible to use PRS test results combined with other risk factors within combined risk prediction models such as CanRisk [50].

To date, wide-scale adoption of PRSs in clinical practice has been hampered by (1) concerns about increased health disparities due to their reduced performance in individuals with non-European genetic descent [53]; (2) debated clinical utility, given their limited predictive values compared to classical diagnostic tests designed to detect already manifested pathologies [72]; and (3) a lack of commercially available regulatorily compliant tests. Considering the advances made in addressing these issues and the rapid evolution of methods to improve PRSs’ transferability across populations [73], while acknowledging that achieving a complete solution to the problems will be a gradual and ongoing process with no end in sight, the authors feel confident that PRSs should currently be considered in the following clinical circumstances.

### 3.1. Personalised Breast Cancer Risk-Based Management of Cancer-Free Women with a Family History of Cancer in Hereditary Cancer Clinics

#### 3.1.1. Women with Negative Breast Cancer MPV Test Findings

Family history is an essential factor guiding screening strategies of family members of breast cancer patients. Testing of rare pathogenic variants is already standard practice for women with significant breast and ovarian cancer family history or known diagnosed MPVs in relatives, and with already demonstrated clinical benefit. Approaches in the case of MPVs are summarized in different guidelines and include earlier more intense screening methods (yearly mammography and MRI), but also risk-reducing surgeries [23]. However, for women in whom MPV testing did not detect the presence of MPVs in them or their families, PRS testing is recommended to fully assess their genetic risk [57,58,59,60,74]. PRSs identify women at a genetically higher risk of breast cancer who tested negative for monogenic risk genes [75].

A study by Evans et al. demonstrated that PRSs gave additional risk modification information for women with familial breast cancer history who received combined a risk estimation using the Tyrer–Cuzick model [56]. The authors concluded that PRSs could enhance risk assessment for women with an increased familial risk who test negative for, or have a low likelihood of, BRCA1/2 mutations [56]. Similarly, a study by Dite et al. examined the extent to which breast cancer risk prediction models could be improved by incorporating known susceptibility SNPs. The study showed that for women under the age of 50, according to the population-based Australian Breast Cancer Family Registry data, a 77 SNP-based PRS improved risk prediction by >20% in combined risk prediction models [58]. A study by Li et al. examined the utility of PRSs in familial but non-BRCA-associated breast cancer cases, demonstrating that according to their PRS-based predicted risk, management for up to 23% of women could be altered [59]. A study by Mars et al. demonstrated that PRSs enhanced risk assessment for first-degree relatives of women with breast cancer, with particularly strong stratification in cases of early-onset family history [61]. Lakeman et al. demonstrated that incorporating PRSs into the BOADICEA combined risk model for family-based risk prediction led to changes in screening recommendations in up to 27%, 36%, and 34% of cases, according to breast cancer screening guidelines from the USA, UK, and the Netherlands, respectively [60]. Stiller et al. evaluated the clinical utility of incorporating PRSs into breast cancer risk assessments in a cohort of German women with suspected hereditary breast and ovarian cancer syndrome [62]. The inclusion of PRSs altered risk stratification based on 10-year risk calculations in 13.6% of individuals. Moreover, integrating PRSs into breast cancer risk predictions led to clinically significant changes in 12.0% of cases, influencing prevention recommendations set by the German Consortium for Hereditary Breast and Ovarian Cancer. These findings support the integration of PRSs into genetic counselling to enhance personalised breast cancer risk assessment [76]. A study by Tüchler et al. assessed estimated lifetime risks and estimated 10-year risks of 425 cancer-free women with a family history of cancer. They used the CanRisk model, including germline MPV status, non-genetic risk factors, and a 306 variant-based PRS, and analysed impact to the proportions of women changing country-specific European risk categories for intensified breast screening [63]. The study findings showed that for women with non-informative MPV status, the inclusion of PRSs and non-genetic risk factors changed clinical recommendations in up to 31.0% of cases, whereas for women who tested negative for a MPV observed in their family, clinical recommendations changed in up to 16.7% of cases. This study provided additional rationale for considering PRSs and non-genetic risk factors for individualized breast cancer risk prediction in routine clinical care [63].

These data show that for women with a family history of breast cancer, but with negative findings in MPV testing, individual risk assessments and corresponding clinical recommendations are incomplete without PRS information and therefore not the best clinical practice anymore.

#### 3.1.2. Women with Breast Cancer MPV Findings

PRSs have been shown in several studies to modify risk evaluations associated with MPVs in high- and moderate-risk breast cancer genes. Incorporating PRSs into genetic testing for MPVs can improve the accuracy of risk estimation and aid risk management decisions for women who are MPV carriers, especially for MPVs in the moderate-risk genes *ATM* and *CHEK2* [64,65,66].

A study by Kuchenbaecker et al. showed that breast cancer PRSs are predictive of cancer risk in *BRCA1* and *BRCA2* carriers and that the incorporation of a PRS into risk prediction models better informs decisions on cancer risk management and increased the accuracy of risk estimation for individuals who inherit an MPV [64]. A study by Gallagher et al. demonstrated that an 86-SNP PRS modified the breast cancer risk for carriers of *BRCA1*, *BRCA2*, *CHEK2*, *ATM*, and *PALB2* MPVs [65]. Gao et al. demonstrated that PRSs enhanced the personalization of breast cancer risk assessment among carriers of MPVs in predisposition genes. Integrating PRSs into risk estimation helped identify over 30% of CHEK2 and nearly half of ATM carriers as having a lifetime risk below 20%, suggesting that PRSs could reduce unnecessary screening and enable more tailored risk management strategies [66]. Similarly, Mars et al. investigated how PRSs modify breast cancer risk in mutation carriers [61]. For both PALB2 and CHEK2, a high PRS was associated with an increased risk of breast cancer. Among women with a PALB2 mutation, those with an average PRS (10th–90th percentile) had a lifetime risk of 55.3% (95% CI: 49.4–61.2%) by age 80. This risk rose to 83.9% (71.2–96.6%) for those with a high PRS (>90th percentile) and decreased to 49.1% (30.6–67.6%) for those with a low PRS (<10th percentile). Women with *CHEK2* and an average PRS had a lifetime risk of 29.3% (95% CI 26.8–31.8%), which doubled to 59.2% (52.1–66.3%) in women with a high PRS and decreased to 9.3% (4.5–14.1%) in women with a low PRS [61]. A study by Lakeman et al. showed that among carriers of MPVs in known moderate breast cancer susceptibility genes, the PRS had a higher impact on *CHEK2* and *ATM* than in the high-risk MPVs [60]. A study by Schreurs et al. analysed the changes in surveillance category by adding a polygenic risk score based on 311 breast cancer-associated variants (PRS311), questionnaire-based risk factors, and breast density on personalised breast cancer risk in unaffected women from Dutch *CHEK2* c.1100delC families [67]. The surveillance advice was reclassified in 20 (34.5%) heterozygotes and 21 (35.6%) non-carriers after adding PRS311. Overall, most heterozygotes were reclassified to a less intensive surveillance, while non-carriers would require intensified surveillance. The addition of PRS, questionnaire-based risk factors, and breast density to family history resulted in more personalised breast cancer surveillance advice in *CHEK2*-families, which may lead to more efficient use of surveillance [67]. In the current Cancer Research UK-funded study called Precision HBOC, a SNP313 PRS is being used to stratify risk in *BRCA1* and *BRCA2* carriers [77].

Summarising the data, we can conclude that the addition of PRS impact gives additional information for more informed decisions regarding the management of breast cancer risk from MPVs, especially in the case of MPVs in the moderate-risk genes *CHEK2* and *ATM*.

### 3.2. Individual Personalised Breast Cancer Prevention and Screening

Many healthcare providers and wellness programs offer more comprehensive and personalised health controls and monitoring than the usual population-based public screening programs. Additionally, such programs are also implemented by employer organizations (corporate wellness). As breast cancer is the most common malignancy among women, the screening and prevention of breast cancer might be included in these services. Consideration of genetic risks, including MPV and PRS testing, may be an important part of such services to increase the precision level of relevant clinical recommendations. A PRS is transmitted according to the principles of polygenic inheritance but additionally confers a risk component independent of family history [78]. Whilst MPVs on a population basis only substantially impact risk in the 1.7% of women who carry them, significantly more women get a meaningful change in risk from a PRS [51]. Breast cancer individual personalised prevention and screening using a PRS is implemented currently in Estonian and United Kingdom private healthcare [44].

### 3.3. Enhancement of Systematic Public Breast Cancer Screening Programs

Screening with mammography reduces breast cancer mortality by 20–40% [6,7,8]. Current population-based breast cancer screening programs are based on age only. The European Commission Initiative on Breast Cancer (ECIBC) gives recommendations for breast cancer screening in women with an average risk of breast cancer, but each country has their own program [10,79]. The current European guidelines recommend for breast cancer screening are as follows [10]:

1. Women aged 40–44: no screening;
2. Women aged 45–49: screening every 2 or 3 years;
3. Women aged 50–69: screening every 2 years;
4. Women aged 70–74: screening every 3 years.

However, breast cancer in younger women tends to be more aggressive, with higher rates of metastasis and poorer survival rates compared to older women with breast cancer. Therefore, early detection and diagnosis may be particularly important for this age group.

Age-based population screening overlooks younger individuals whose risk levels already exceed the thresholds for programme entry, such as women with elevated genetic risk who may develop breast cancer earlier than the designated screening age. Implementing personalised risk assessments is essential to identify those who would benefit from earlier screening initiation. Additionally, for high-risk women over age 50, current screening with 2-year intervals might not be considered optimal and miss many interval cases that would otherwise be detected earlier. Therefore, breast cancer screening might benefit from a better adaptation to the individual risk level of a woman.

PRSs enable the stratification of women into different risk categories, allowing for tailored recommendations on the timing of mammography screening and other preventive measures [51,52,55,80]. They help identify women at higher genetic risk who reach the threshold for population screening at a younger age, comparable to the risk level of a 50-year-old woman eligible for routine screening.

Wolfson et al. suggest based on their study data that population-wide breast cancer screening programmes aiming to stratify women by genetic risk should prioritize PRSs over rarer but highly penetrant variants or family history [34]. An analysis by Lee et al. demonstrated that PRSs are a stronger independent breast cancer risk factor than family history or mammographic density [35]. This finding is supported by other studies [81,82]. Van den Broek et al. conducted a comprehensive analysis assessing the clinical utility of PRSs and first-degree family history of breast cancer in guiding screening decisions for women aged 30–50 years [54]. Their findings suggest that combining PRSs with family history could help refine screening recommendations before age 50 for women at elevated risk. However, this approach is expected to increase overdiagnoses and false positives. Compared to biennial screening from ages 50 to 74, the combined use of PRSs and family history resulted in the greatest improvements, with a 29% increase in life-years gained and an 18% reduction in breast cancer mortality [54]. The benefits of earlier screening rose significantly as PRSs increased—women with a three times or higher risk than average could begin screening at 30 or 35 years, while those at greater-than-average risk but below this threshold could start at 40 years. Conversely, those in the lowest risk category could be screened triennially between ages 50 and 74 [54]. Mars et al. examined the role of PRSs, family history, and MPVs in stratified breast cancer screening [68]. Using FinnGen data (N = 117,252) linked to the Mass Screening Registry, the study compared the predictive performance of PRSs with family history and MPVs in moderate-risk (CHEK2) and high-risk (PALB2) susceptibility genes. Women with a high PRS faced an increased risk of interval breast cancers, whereas those with a low PRS had a reduced likelihood of both interval and screen-detected breast cancers. By leveraging real-world screening data, the study demonstrated the effectiveness of PRSs for risk stratification, both independently and in combination with family history and MPVs [68]. A modelling analysis by Huntley et al. has shown that under favourable assumptions, the use of PRSs in UK cancer screening suggests a modest potential efficiency gain in breast cancer case detection and deaths averted [69]. Bolze et al. investigated breast cancer incidence and age of onset among women with low PRS risk compared to those at average risk, assessing the feasibility of delaying mammography based on genetic risk stratification [83]. In a case-control study involving 25,591 women, 9.1% were identified as having a low genetic risk for breast cancer. These women developed breast cancer significantly later than those at average or high risk, suggesting that mammography screening could potentially be deferred by 5 to 10 years, optimizing healthcare resource allocation [83].

Personalised breast cancer screening based on hereditary risks for women at age 35–49 has been tested in the Estonian branch of the BRIGHT project using the family cancer history questionnaire, the PRS test AnteBC risk estimates for all women, and MPV testing based on family cancer history [84]. Amongst 799 participants, 90 (11.3%) had MPVs tested after consultations by clinical geneticists, resulting in 4 (4.4%) MPV diagnoses. PRS testing of all participants identified 330 (41.3%) women with elevated polygenic risk, with 124 (37.6%) women already at higher risk than the average 50-year-old [84]. The BRIGHT study demonstrated the feasibility of genetics-based precision prevention, facilitating earlier BC screening for younger women with elevated genetic risks. The predominantly digital service minimised the burden on healthcare personnel [84]. The BRIGHT study also assessed the cost-effectiveness of risk-stratified breast cancer screening in Estonia for women starting at age 35 versus standard mammography screening for ages 50–69, focusing on the PRS test’s isolated impact [85]. Risk-stratified screening led to a redistribution of breast cancer stages, with more early stages (0–I) and fewer advanced stages (II–IV), and averted 1.5 breast cancer deaths per 1000 women screened. Risk-stratified screening resulted in larger net costs of €145,235 (mainly related to PRS test and counselling costs), and a gain of 3.85 QALYs, with an ICER of €37,755 per QALY gained [85]. The conclusion was that PRS-tailored breast cancer screening has a clinical benefit and is cost-effective in Estonia.

The accumulated evidence clearly shows that age-based screening is probably not the best solution, and screening could rather be organized based on women’s individual risks. For this purpose, PRSs, family history of cancer, MPV testing and, if feasible, the use of combined risk assessment models are important components of risk assessment. Risk-based approaches must also take into account the ethnic background and the availability of current evidence in both the PRS and combined risk assessment aspects.

## 4. Possibilities for Clinical Recommendations for Personalised Prevention and Screening of Breast Cancer Based on PRS Results

There are three key foundations for implementing clinical recommendations based on PRSs, as outlined below. In the article on the development and clinical application of a breast cancer PRS test, authors have described the PRS test output, including the z-score (expressed as standard deviations), risk percentile, relative risk compared to individuals of the same age and population, and the corresponding 10-year absolute risk [44]. Authors also outlined a version of clinical recommendations for PRS risk-based breast cancer prevention and screening. These recommendations may be adapted to align with existing country-specific guidelines for risk-based approaches.

### 4.1. Comparison with the Average Risk of the Same Population at the Same Age, Combined with a Comparison to the Average Risk upon Initiation of Mammographic Screening

**Figure 1:**
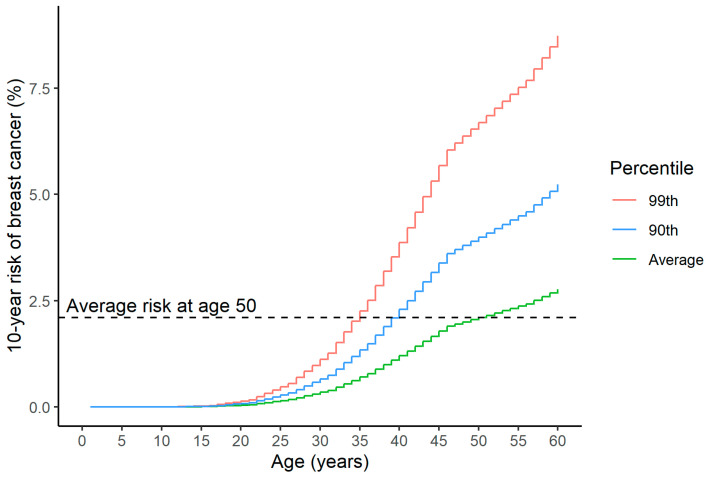
The 10-year risk of developing breast cancer according to a woman’s age and PRS2803 percentile [44]: 99th percentile—red, 90th percentile—blue, population average—green. Population background is from breast cancer incidence data for Norway 2018–2020 (from Nordcan 2.0).

Principally, societies have agreed that the average risk level at age 50 in most European countries is suitable to start public mammography screening [79]. It may be logical to start screening at a younger age for women if their PRS-driven risk level achieves the same level or is higher than the average risk at age 50. This is according to principles of equitability and equivalence of risks. Figure 1 describes the age difference for women at high PRS risk in reaching same risk level compared to women at average risk at baseline for the start of current screening (age 50).

The WISDOM study applies this approach to women aged 40 to 49 years, recommending screening when their five-year risk meets or exceeds that of the average 50-year-old woman [86]. This study employs five-year risk thresholds, as screening and preventive measures are most effective for individuals at imminent risk of developing cancer. Additionally, five-year risk estimates are commonly used to guide chemoprevention strategies. In the WISDOM study, the five-year risk threshold for women aged 50 was set at 1.3%.

### 4.2. Comparison with Similar Risk MPVs

**Figure 2:**
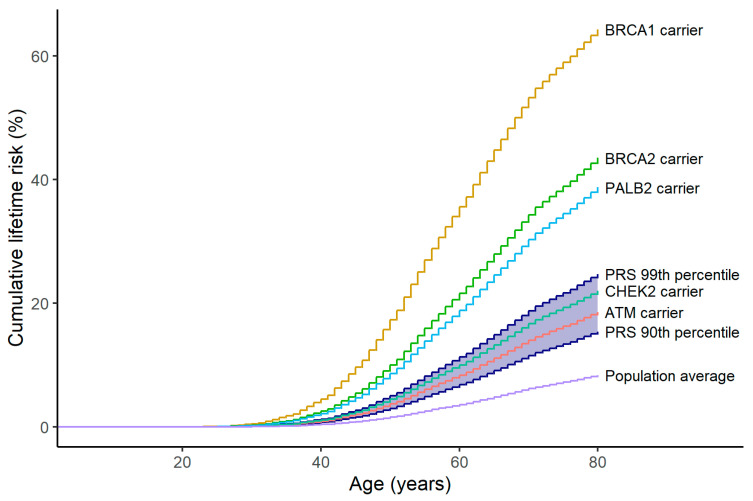
Cumulative lifetime breast cancer risks of carriers of protein-truncating pathogenic variants in breast cancer risk genes compared to women in the PRS2803 upper decile (blue). Breast cancer risks of pathogenic variant carriers are from the analysis by the Breast Cancer Association Consortium [18]. Population background is from breast cancer incidence data for Norway 2018–2020 (from Nordcan 2.0).

Like elevated polygenic risk, a moderately elevated risk level (lifetime risks 25–30%) applies to MPVs in the genes *ATM* and *CHEK2* [23], as shown in Figure 2. Accordingly, on the same PRS risk level, similar clinical recommendations can be given as in the case of moderate-risk MPVs. A comparative modelling analysis has shown that for women with *ATM*, *CHEK2*, and *PALB2* pathogenic variants, annual MRI screening starting at 30 to 35 years followed by annual MRI and mammography at 40 years may reduce breast cancer mortality by more than 50% [87]. A similar approach is feasible for women at the same risk level using PRS testing.

Breast cancer risk management in the case of moderate-risk MPVs is included, for example, in the NCCN guidelines. The NCCN Guidelines for Genetic/Familial High-Risk Assessment: Breast, Ovarian, and Pancreatic, Version 3.2024 [88] state that “In the case of MPVs in *ATM* and *CHEK2* is recommended annual mammography at age 40 years and consider MRI with contrast starting at age 30–53”.

### 4.3. Comparison with Already Existing National Guidelines Based on Other Risk Factors (Not Including PRSs) for Risk-Stratified Breast Cancer Screening According to Different Risk Levels

In this context, it is possible to use PRS isolated risk information as well as total risk estimation using combined models. The current guidelines for breast risk-stratified screening and surveillance of selected countries are reflected below, primarily based on the current practice of the authors of this article.

#### 4.3.1. Guidelines in the United Kingdom

The UK National Institute for Health and Care Excellence (NICE) guidelines for the management of women with familial breast cancer risk are using the thresholds for risk categories [22] shown in Table 1.

The NICE guidelines classify breast cancer risk into three categories: general population risk, moderate risk, and high risk [22]. Women at general population risk have an estimated lifetime breast cancer risk of approximately 11% [89]. Those at moderate risk have a lifetime risk exceeding 17% but below 30%, while women at high risk have a 30% or greater likelihood of developing breast cancer over their lifetime. Accordingly, the UK NICE guidelines have defined a moderate risk as 1.5 to 2.7 times higher than average risk and a high risk as more than 2.7 times higher than average.

Breast cancer PRSs can allocate risk groups based on this accordingly [44]:

1. General population risk: 1.–79. percentiles;
2. Moderate risk: 80.–97. percentiles;
3. High risk: 98.–99. percentiles.

The NICE guidelines also give recommendations on surveillance for high- and moderate-risk groups of different ages, recommending annual mammography from age 40 for increased-risk groups.

#### 4.3.2. Guidelines in Germany

German guidelines for breast cancer management, including screening, are characterized in “Interdisziplinäre S3-Leitlinie für die Früherkennung, Diagnostik, Therapie und Nachsorge des Mammakarzinoms” [90]. The current German breast cancer guidelines recommend that women aged 40–49 undergo a mammogram screening every two years, but the guidelines suggest that the decision to undergo mammography screening in this age group should be based on an individual assessment of the potential benefits and harms, taking into account the woman’s personal risk factors for breast cancer and her preferences [90]. There are in place principles and guidelines for “Breast Cancer Risk, Genetics and Prevention” by Arbaitsgemeinschaft Gynäkologische Onkologie (AGO) [91].

In Germany, genetic testing for breast cancer is primarily offered to individuals with a personal or family history of breast or ovarian cancer. The current guidelines for genetic testing in Germany are based on recommendations from the German Society of Human Genetics (GfH) and the German Consortium for Hereditary Breast and Ovarian Cancer (GC-HBOC)—Deutsches Konsortium Familiärer Brust- und Eierstockkrebs [92]. The GC-HBOC recommends, for moderate MPV carriers’ annual clinical examination, breast ultrasonography and MRI from age 30 and mammography annually or biannually from age 40.

#### 4.3.3. Guidelines in Norway

In Norway, national guidelines and recommendations exist for risk-stratified BC prevention [93]. Women with a family history of breast cancer and considered being at increased risk after assessment by medical geneticists, with no pathogenic variants in high-risk breast cancer genes, are offered annual two-plane mammography from the age of 40–60. In families with cases of breast cancer before the age of 40, it may be considered to start mammography checks from the age of 30 [93]. The inclusion of PRS information may add value and give possibilities for a more systematic implementation of risk-stratified prevention and screening.

#### 4.3.4. Guidelines in Sweden

In Sweden, the National Board of Health and Welfare recommends population-based mammography screening for ages 40–74 with screening intervals of 18 to 24 months [26]. For people with a hereditary increased risk, other intervals or methods may be relevant [26]. Swedish guidelines recommend MPV testing for cancer-free women according to family history, but the risk of primary breast cancer in a woman with healthy breasts can also be roughly estimated using epidemiological risk models. If applicable, the CanRisk model is recommended in the first place [26]. For women with moderately increased risk (based on the presence of pathogenic variant in gene associated with moderately increased risk, or alternatively women with an epidemiologically greater than 20% lifetime risk), annual imaging (breast) from about 5 years before the lowest age of onset in the family or at the latest from the age of 40 up to about the age of 74 is recommended [26].

#### 4.3.5. Guidelines in Portugal

According to the National Screening Program in Portugal, 50–69-year-old women are invited to breast cancer screening every two years [94]. The national guidelines for MPV testing for women without a cancer diagnosis are based on specific criteria that focus on identifying those at higher risk due to family history or other factors [94]. These guidelines are intended to identify individuals who may benefit most from genetic testing due to an increased risk of hereditary breast cancer, allowing for personalised risk management and preventive strategies. Women with high-risk breast cancer MPVs (e.g., BRCA1 and BRCA2), moderate-risk MPVs (e.g., CHEK2, ATM, and PALB2), or other increased risk factors, such as a strong family history or previous breast conditions, are advised to start screening earlier, typically around 30–40 years, with annual mammograms and breast MRI depending on their specific risk profile. These women receive genetic counselling and personalised management plans.

#### 4.3.6. Guidelines in Estonia

In Estonia, women aged 50–74 years are invited to breast cancer screening at two-year intervals. Risk-based management of hereditary cases is performed according to healthcare institution internal guidelines. Estonia has conducted a national clinical feasibility project investigating the implementation of PRS- and MPV-based breast cancer risk-stratified screening for women younger than current standard screening programme [95]. Breast cancer PRS testing for cancer-free women is currently implemented in healthcare services [44]. The Estonian Health Insurance Fund is preparing a PRS-based, risk-stratified screening for women at age 40; the programme is aimed to start early 2025 [96].

## 5. The Regulatory and Legal Status of Breast Cancer Risk Estimation Tools in the European Union in the Context of Polygenic Risk Score Testing

As the regulatory understanding of PRSs in the scientific literature and clinical practice seems somewhat unclear, we also examine the current regulatory background in the European Union. PRS tests for disease predispositions are regulated under the EU In Vitro Diagnostic Regulation (IVDR) 2017/746 because they are considered in vitro diagnostic (IVD) medical devices [97]. According to the regulation, an IVD medical device is any device which, whether used alone or in combination, is intended by the manufacturer for the in vitro examination of specimens derived from the human body solely or principally to provide information on a physiological or pathological state, or a congenital abnormality, or to monitor therapeutic measures. Accordingly, PRS tests are classified as in vitro diagnostic (IVD) medical devices and must comply with the EU’s IVDR 2017/746. These tests must demonstrate clinical validity and utility, with a clear demonstration of performance characteristics and safety. They are subject to pre-market scrutiny and must fulfil post-market surveillance obligations. Providers must ensure the tests are not discriminatory and consider the diversity of the population, including genetic variations across different ancestries.

Another option in the EU is to perform PRS tests as Laboratory Developed Tests (LDTs) under EU IVDR 2017/746 with relevant restrictions [97]. The IVDR imposes more stringent requirements on LDTs than the previous directive, aiming to align their safety and performance standards with commercially available IVDs. There is a health institution exemption under Article 5(5), but it requires LDTs to be justified, documented, and notified to national competent authorities. LDTs must be manufactured and used within the same EU member state health institution, under a quality management system. Performance evaluations and compliance with the general safety and performance requirements are mandatory. These regulations ensure a high standard of quality and safety for diagnostic tests, including PRS tests, whether they are commercial kits or developed in-house within EU health institutions. Lab-developed tests cannot be used for public screening programs (on an industrial scale).

## 6. Conclusions

In conclusion, the adoption of breast cancer PRS testing represents a significant advancement in personalised medicine, namely in personalised screening. By incorporating PRSs into clinical practice, healthcare providers can offer more precise risk assessments, tailored prevention strategies, and optimized screening programs, ultimately improving patient outcomes and enhancing the efficiency of breast cancer care.

## Figures and Tables

**Table 1:** Breast cancer risk categories by the UK NICE guidelines [22], with corresponding relative risks added based on average lifetime risk data [89].

	Breast Cancer Risk Category		
	Near Population Risk	Moderate Risk	High Risk
Lifetime risk from age 20	Less than 17%	17% or greater but less than 30%	30% or greater
Risk between ages 40 and 50	Less than 3%	3–8%	Greater than 8%
Corresponding relative risk	Less than 1.5	1.5–2.7	Greater than 2.7
